# Metabolic Response of Human Osteoarthritic Cartilage to Biochemically Characterized Collagen Hydrolysates

**DOI:** 10.3390/ijms18010207

**Published:** 2017-01-20

**Authors:** Saskia Schadow, Viktor S. Simons, Guenter Lochnit, Jens Kordelle, Zuzana Gazova, Hans-Christian Siebert, Juergen Steinmeyer

**Affiliations:** 1 Laboratory for Experimental Orthopaedics, Department of Orthopaedics, Justus Liebig University Giessen, Paul-Meimberg-Str. 3, 35392 Giessen, Germany; saskia.schadow@meckit.de (S.S.); Viktor.S.Simons@med.uni-giessen.de (V.S.S.); jens.kordelle@ekm-gi.de (J.K.); 2 Protein Analytics, Department of Biochemistry, Faculty of Medicine, Justus Liebig University Giessen, Friedrichstr. 24, 35392 Giessen, Germany; Guenter.Lochnit@biochemie.med.uni-giessen.de; 3 Agaplesion Evangelical Hospital Mittelhessen, Paul-Zipp-Str. 171, 35398 Giessen, Germany; 4 Institute of Experimental Physics, Department of Biophysics, Slovak Academy of Sciences, Watsonova 47, 040 01 Košice, Slovakia; gazova@saske.sk; 5 RI-B-NT Research Institute of Bioinformatics and Nanotechnology, Franziusallee 177, 24148 Kiel, Germany; hcsiebert@aol.com

**Keywords:** collagen hydrolysate, osteoarthritis, cartilage, ADAMTS, MMP, TIMP, IL6, MALDI-TOF, Peptan^®^ F, Mobiforte^®^

## Abstract

The most frequent disease of the locomotor system is osteoarthritis (OA), which, as a chronic joint disease, might benefit more from nutrition than acute illnesses. Collagen hydrolysates (CHs) are peptidic mixtures that are often used as nutraceuticals for OA. Three CHs were characterized biochemically and pharmacologically. Our biophysical (MALDI-TOF-MS, NMR, AFM) and fluorescence assays revealed marked differences between CHs of fish (Peptan^®^ F 5000, Peptan^®^ F 2000) and porcine (Mobiforte^®^) origin with respect to the total number of peptides and common peptides between them. Using a novel dual radiolabeling procedure, no CH modulated collagen biosynthesis in human knee cartilage explants. Peptan^®^ F 2000 enhanced the activities of the aggrecanase ADMATS4 and ADMATS5 in vitro without loss of proteoglycan from cartilage explants; the opposite effect was observed with Mobiforte^®^. Interleukin (IL)-6, matrix metalloproteinase (MMP)-1, -3 and -13 levels were elevated in explants that were treated with Mobiforte^®^ and Peptan^®^ F 5000, but not with Peptan^®^ F 2000. In conclusion, the heterogeneous peptide composition and disparate pharmacological effects between CHs suggest that the effect of a CH preparation cannot be extrapolated to other formulations. Thus, the declaration of a CH as a safe and effective nutraceutical requires a thorough examination of its pleiotropic effects.

## 1. Introduction

Osteoarthritis (OA) is a widespread joint disease that most often leads to long-term immobility of persons aged over 65 years. That OA is no longer an inevitability of old age has created significant demand for its prevention and therapy. Such compounds should be effective with few or no side effects with long-term use, despite age-conditioned accompanying illnesses and other medication use. The primary aim of current drug therapy for OA is to inhibit pain and inflammation [1]. However, the lack of a cure for joint destruction in OA underscores the need for safe and effective secondary and tertiary preventive measures against the progressive damage to cartilage during OA.

OA might be prevented by food factors, which has created significant public interest concerning the relationship between food, nutritional supplements and OA. As a chronic disease, OA can likely benefit from food supplements compared with acute illnesses. This is caused by the small effects of food factors on biological targets, such that clinically-relevant effects are achieved only after longer treatment periods. Food supplements, such as Collagen hydrolysates (CHs), are often marketed extensively, whereas only limited data on their safety, efficacy and mode of action exist [2,3,4,5,6].

CHs have been tested as biopolymers for biomedical, medical and nutritional applications [3,4,5,6,7,8,9,10]. Most collagen is derived from enzymatic hydrolysis of cow and pig skin, but non-mammalian collagen from fish is an alternative source, based on its high availability and the lack of religious restrictions on its use. Byproducts of fish, such as skin, fins, scales and bones, are normally discarded as waste [7]. Naturally, vertebrates express over 28 types of collagen. Thus, the peptide composition of byproducts from fisheries might be influenced by the species and during their conversion to suitable biomedical, pharmaceutical and nutritional products.

CHs initially received tremendous public attention, based on in vitro observations that a bovine-derived peptide mixture stimulated proteoglycan and collagen synthesis in cultured bovine chondrocytes [11]. These data suggested that it is possible to slow damage to the extracellular matrix in OA using a collagenous peptide mixture. However, in a subsequent experiment, none of three bovine type I CHs—CH-Alpha^®^, and Peptan^®^ B 2000 and 5000—effected type II collagen biosynthesis of human cartilage [2].

Fish CH (Norland) from the skin of cod, haddock and pollock was reported to induce chondrogenic differentiation in equine stromal cells from fatty tissue [12] and in rat mesenchymal stem cells from bone marrow [13], whereas CH from silver carp increased the collagen content of human fibroblasts from gingiva [14]. We have observed weak binding of small collagen peptides to the α2A-domain of the integrin receptor, which might constitute a mechanism of these effects [15,16].

Absorption of CHs from the gastrointestinal tract is a prerequisite for any in vivo effects to occur after oral application, and intestinal absorption of acid- or pepsin-soluble type II CH has been reported in an in vitro gut sac experiment, in which peptides, ranging from 8–70 kDa, were observed on the serosal side of the intestine [17]. Furthermore, mice absorb radiolabeled type I collagen hydrolysate through their intestine, and some radioactivity was recovered in joints [18].

The destruction of the extracellular matrix of articular cartilage is a hallmark of OA and occurs through the coordinated activities of aggrecanases and matrix metalloproteinases (MMPs). The aggrecanases ADAMTS4 and ADAMTS5 (a disintegrin and metalloproteinase with a thrombospondin motif) are crucial enzymes in the initial proteolytic degradation of proteoglycans, of which ADAMTS4 is induced by proinflammatory cytokines and ADAMTS5 is constitutively expressed [19,20].

Significant structural differences and similarities exist between the closely-related ADAMTS4 and -5 with regard to their active sites, which are also the binding sites for inhibitors of these enzymes. The overall structures of ADAMTS4 and ADAMTS5, which have 48% sequence identity outside of the pre- and pro-domains, show a common fold containing the N-terminal metalloprotease domain and the C-terminal disintegrin-like domain [21]. The metalloprotease catalytic domains, comprising residues 214–428 in ADAMTS4 and residues 264–476 in ADAMTS5, consist of the active site, two calcium-binding sites and catalytic zinc.

Compared with other MMPs, the active sites in ADAMTS4 and ADAMTS5 are flexible, showing a high degree of conformational freedom [21,22,23,24]. The conformational mobility of the active sites of ADAMTS4 and ADAMTS5 is another property of mature aggrecanases, which might be needed for high-affinity association with a substrate and related to the dynamic behavior of proteoglycans.

The initial degradation of proteoglycans by ADAMTS4 and ADAMTS5 is continued by additional proteolytic digestion by MMP-3, for example. Further, MMP-3, with MMP-1 and MMP-13, degrades collagen in the extracellular matrix of cartilage [20,25,26]. Tissue inhibitors of metalloproteinases (TIMP)-1–4 regulate the activity of MMPs and aggrecanases, of which TIMP-3 inhibits ADAMTS4 and ADAMTS5 [27]. Conversely, fragments from collagen type II, extracted from human cartilage, accelerate damage to the extracellular matrix and upregulate MMPs. These events contribute to the destruction of cartilage during OA or might be processes in normal metabolic feedback [28,29].

Inflammation and progressive joint destruction are related to OA, for which several mediators have been identified. IL-1β, an inflammatory factor, stimulates the biosynthesis of catabolic proteases and impedes the generation of proteoglycans and collagen by chondrocytes [25,26]. Furthermore, other inflammatory mediators, such as IL-6 and NO, are linked to OA and cytokine-induced pathways [25,26,30]. IL-6 is produced in the synovium during synovitis, and serum IL-6 levels have been associated with knee pain in the early and advanced stages of OA [31,32], cartilage loss [33] and the likelihood of radiologically-discernable knee OA [34]. Notably, IL-6, only with its soluble receptor (sIL-6R), synergizes with IL-1, TNFα and mechanical injury to induce collagen and proteoglycan loss from bovine and human cartilage [35,36,37]. The IL6/sIL-6R complex activates gp130 on cells and is competitively inhibited by soluble glycoprotein 130 (sgp130), blocking IL-6 trans-signaling [38,39]. Thus, the IL-6 trans-signaling pathway is another target for future therapeutic strategies, but the effects of CH on this pathway have not been reported so far.

In searching for CHs that stimulate collagen biosynthesis and antagonize catabolic and inflammatory mechanisms that are involved in the destruction of human articular cartilage, we analyzed commercially available fish and porcine CHs. Specifically, we examined whether these CHs: (a) modulate the synthesis of type II collagen and inhibit the loss of proteoglycan from human OA knee cartilage; (b) affect IL-6 trans-signaling and the aggrecanases and MMPs that mediate the degradation of cartilage in OA; and (c) determine their peptide composition. Based on our comprehensive biochemical and metabolic analysis, it was concluded that the investigated CHs markedly differ both concerning their peptidic composition, as well as their effects on cartilage metabolism.

## 2. Results

### 2.1. MALDI-TOF-MS Analysis of CHs

**Figure 1:**
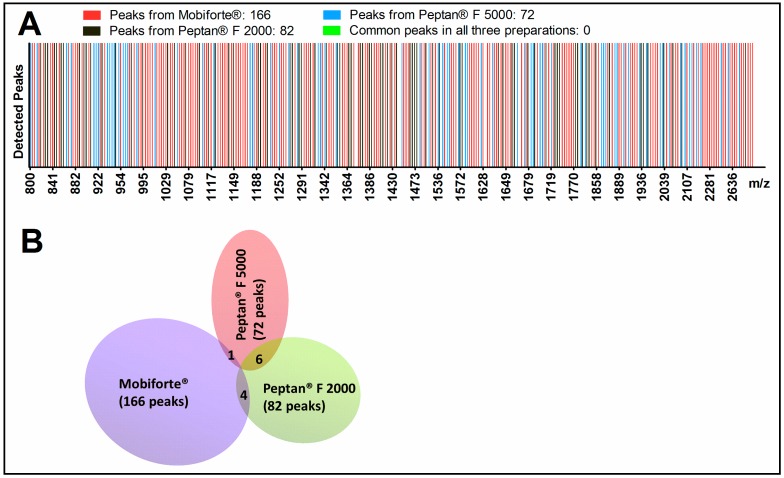
Mass spectra from CHs by MALDI-TOF-MS. Representation of mass spectra from Mobiforte^®^, Peptan^®^ F 5000 and Peptan^®^ F 2000 as a (**A**) gel-like view; and (**B**) Venn diagram. MALDI-TOF-MS analysis was performed in reflector mode with a mass-to-charge ratio (*m*/*z*) range between 500 and 4000 *m*/*z* using mass gates. The numbers in brackets are the number of total peaks each representing a peptide of each CH. The numbers in the intersections of the Venn diagram are peptides shared by both preparations. No common peptide was identified in all three CHs.

Matrix-assisted laser desorption/ionization (MALDI)-time of flight (TOF)-mass spectrometry (MS) analysis, performed in reflector mode between 500 and 4000 *m*/*z*, revealed marked qualitative differences in peptide composition between the three CHs. As shown in Figure 1A,B, Mobiforte^®^, Peptan^®^ F 2000 and Peptan^®^ F 5000 differed with respect to the total number of peaks in each preparation. We noted significantly more peptides in Mobiforte^®^ versus the two Peptan preparations: 166 in Mobiforte^®^ compared with 72 in Peptan^®^ F 5000 and 82 in Peptan^®^ F 2000. The three CHs also varied, based on a few common peptides between them (Figure 1A,B). For instance, Mobiforte^®^ and Peptan^®^ F 5000 shared one peptide (*m*/*z* 1447), and both CHs from fish (Peptan^®^ F 5000, Peptan^®^ F 2000) had six peptides in common (*m*/*z* 1360, 1572, 1662, 1685, 1953, 2039). No common peaks (0%) were found between all three CHs.

The average molecular weight of Mobiforte^®^, as determined by MALDI-TOF-MS spectra in the linear mode, was 3.120 Da, which is between the values that have been reported by the manufacturer for Peptan^®^ F 2000 (2.000 Da) and Peptan^®^ F 5000 (5.000 Da).

### 2.2. NMR Analysis of CHs

**Figure 2:**
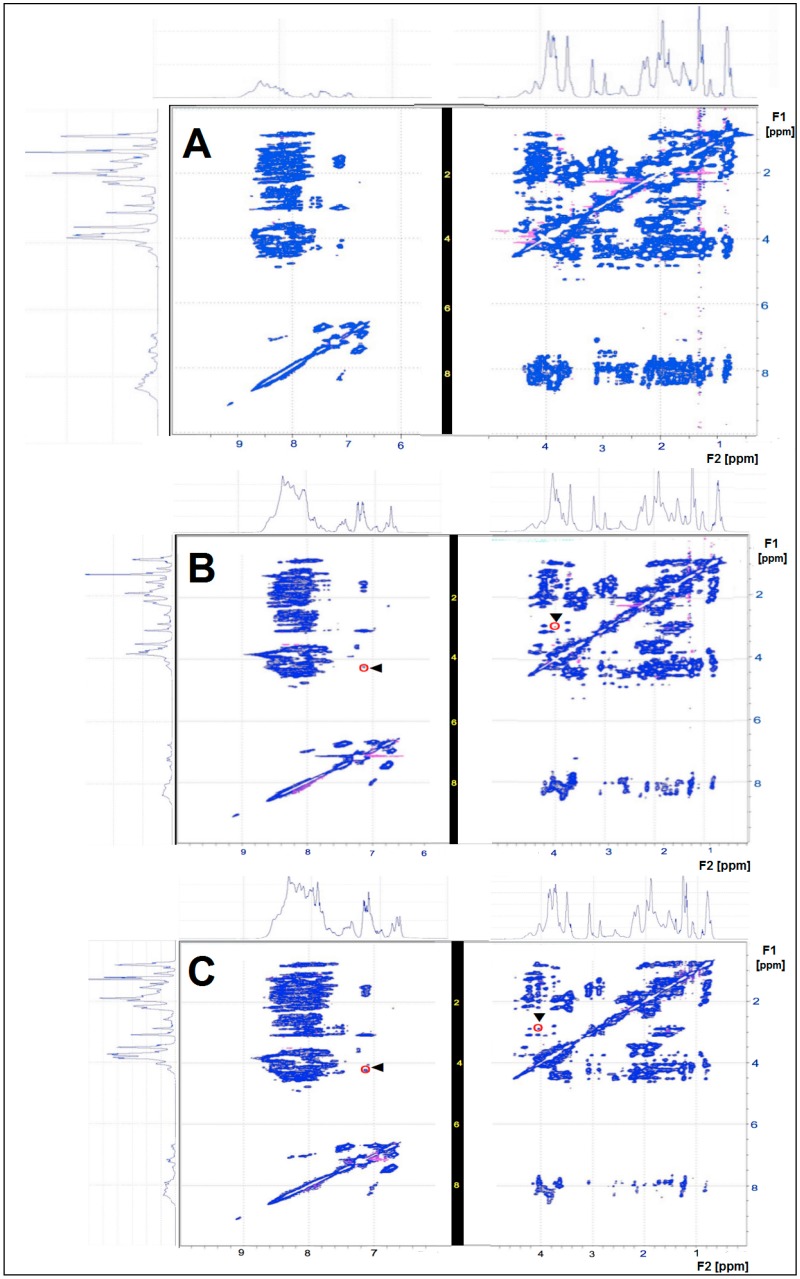
NMR-TOCSY spectra of the three CHs tested. The TOCSY spectra show characteristic signal alterations that can be used to discriminate the three CHs: (**A**) Mobiforte^®^; (**B**) Peptan^®^ F 5000; and (**C**) Peptan^®^ F 2000. Two cross peaks (at 4.3/7.4 ppm and at 3.0/4.0 ppm) in the TOCSY spectra of Peptan^®^ F 5000 and Peptan^®^ F 2000 are highlighted by an arrowhead and red circle. Cross peaks are all signal spots that occur below and above the diagonal line that represents the basic one-dimensional spectrum. The cross peaks below and above the diagonal line occur symmetrically and indicate which protons are coupled, based on their F1 and F2 ppm values.

By nuclear magnetic resonance (NMR)-total correlation spectroscopy (TOCSY), we observed characteristic fingerprint patterns of the CHs (Figure 2), allowing us to discriminate heterogeneous peptide mixtures (Mobiforte^®^, Peptan^®^ F 5000 and Peptan^®^ F 2000) from each other. Figure 2 highlights two cross peaks as an example in the NMR-TOCSY spectra of Peptan^®^ F 5000 and Peptan^®^ F 2000 at 4.3/7.4 ppm and 3.0/4.0 ppm, respectively. When comparing the corresponding peaks between Peptan^®^ F 5000 and Peptan^®^ F 2000 with regard to shape and height, the differences can be used to perform a detailed signal-dependent analysis. The appearance of cross peaks in a NMR-TOCSY spectrum indicates that the corresponding protons at characteristic chemical shift values experience scalar coupling, i.e., coupling via the chemical bonds of covalently-linked protons. The cross peaks below and above the diagonal line occur symmetrically and indicate which protons are coupled, based on their F1 and F2 ppm values (Figure 2). The cross peaks formed fingerprint patterns, clearly differentiating Mobiforte^®^, Peptan^®^ F 5000 and Peptan^®^ F 2000 (Figure 2).

Diffusion ordered spectroscopy (DOSY) is the optimal NMR method for analyzing mixtures of peptides to collect data on the molecular weight distribution. By NMR, the molecular weight (MW) distributions of CHs from Mobiforte^®^, Peptan^®^ F 5000 and Peptan^®^ F 2000 were 3.5–7.4, 31–654 and 6.7–17.9 kDa, respectively. In our DOSY experiments, the MW distribution of the CHs reflected the aggregation behavior of individual CH fragments, resulting in the formation of larger peptide aggregates.

### 2.3. Amyloid Fibrillization and Atomic Force Microscopy of CHs

**Figure 3:**
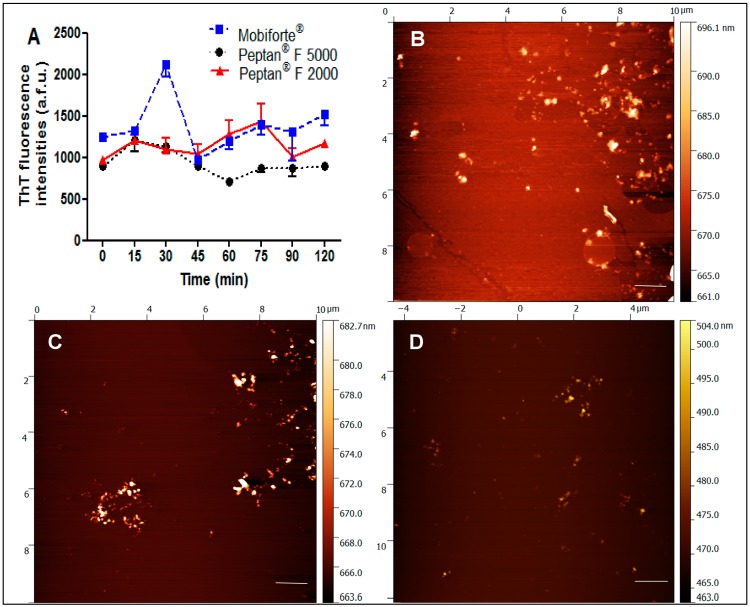
Fibrillization and atomic force microscopy images of CHs. (**A**) Time dependence of fibrillization of three types of CHs monitored by the ThT fluorescence assay. The error bars represent the average deviation for repeat measurements of three separate samples. Peptan^®^ F 5000 (**black circles**), Peptan^®^ F 2000 (**red triangles**) and Mobiforte^®^ (**blue squares**); (**B**–**D**) Atomic force microscopy images of the CHs: Peptan^®^ F 5000 (**B**); Peptan^®^ F 2000 (**C**); and Mobiforte^®^ (**D**). Images were taken after treatment under neutral conditions at pH 6.0 and 65 °C as described in the Materials and Methods. Bars represent 1 µm.

The ability of the three CHs to form amyloid fibrils in nearly neutral conditions (20 mM Na_2_HPO_4_ + 80 mM NaCl buffer, pH 6.0), high temperature and with constant stirring was examined. These conditions promote the fibrillization of proteins [40]. Thioflavin T (ThT) is a specific dye that enhances its fluorescence intensity up to 80,000–90,000 arbitrary fluorescence units (a.f.u.) on binding to amyloid fibrils. As shown in Figure 3A, Peptan® F 5000, Peptan® F 2000 and Mobiforte^®^ had a similar level of ThT fluorescence, with no increase in intensity from the beginning of the process. The recorded fluorescence signals were lying in the range of 700–1500 a.f.u. and correspond to those of non-aggregated proteins. Thus, our results indicate that none of the CHs have the ability to form amyloid fibrils.

Our results were confirmed by direct visualization of the samples by atomic force microscopy (Figure 3B–D). The images of Peptan^®^ F 5000, Peptan^®^ F 2000 and Mobiforte^®^ showed merely the presence of small amorphous aggregates that lacked the fibrillar structure that is typical of amyloid fibrils.

### 2.4. Collagen Synthesis of Human Cartilage Treated with CHs

**Figure 4:**
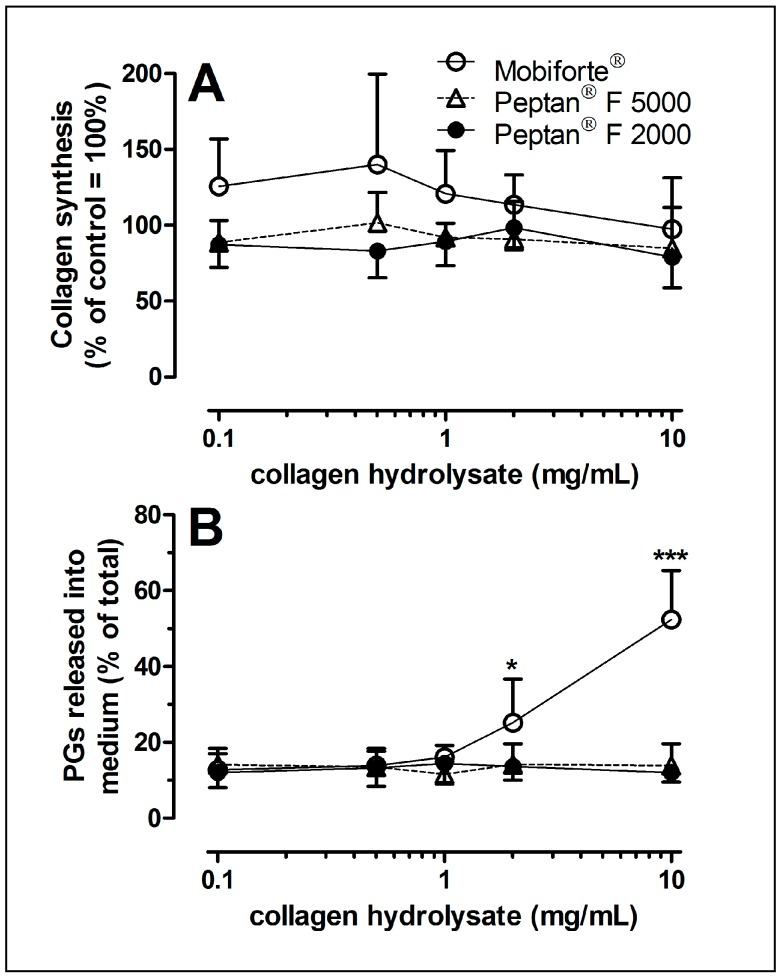
Effect of CHs on collagen synthesis and proteoglycan release from OA cartilage. (**A**) Incorporation of [^3^H]-proline and [^14^C]-proline into collagen of human cartilage explants was determined by measuring the radioactivity in hydroxyproline. The [^14^C/^3^H] incorporation ratio was then calculated and expressed as the percentage of untreated control (100%) in the presence of 0.1, 0.5, 1, 2 and 10 mg/mL of Mobiforte^®^, Peptan^®^ F 5000 or Peptan^®^ F 2000. Data are the mean + SD (*n* = 4–5); (**B**) Proteoglycan loss from cartilage explants into nutrient media in the presence of various concentrations of CHs was determined during the treatment period, lasting six days. Data were calculated as the percentage of total proteoglycans found in the media and corresponding explants (=100%); data shown are the mean + SD (*n* = 6). *p*-values equal or less than 0.05 were considered statistically significant: * 0.01 < *p* ≤ 0.05, *** *p* ≤ 0.001.

Figure 4A shows that Mobiforte^®^, Peptan^®^ F 2000 and Peptan^®^ F 5000 did not stimulate or inhibit collagen synthesis, even over a broad range of concentrations. Although the metabolism of joint cartilage in the early stages of OA differs from that of the late stages [41,42], we have reported similar effects of three bovine CHs on cartilage between early-stage (Collins score < 1.5) and middle-stage disease (Collins score 1.5–3) [2]. Thus, we only analyzed the biosynthesis of collagen using cartilage explants with moderate OA changes.

### 2.5. Proteoglycan Loss from CH-Treated Cartilage Explants

To determine the extent to which CHs modulate the loss of extracellular matrix, explants were exposed to various concentrations of CHs (Figure 4B). Even at a high concentration (10 mg/mL), Peptan^®^ F 2000 and Peptan^®^ F 5000 failed to enhance the release of proteoglycans from the extracellular matrix of explants to the nutrient media. However, 2 and 10 mg/mL Mobiforte^®^ increased the loss of proteoglycan by two-fold and four-fold (Figure 4B).

### 2.6. In Vitro Effects of CHs on Aggrecanase Activity

**Figure 5:**
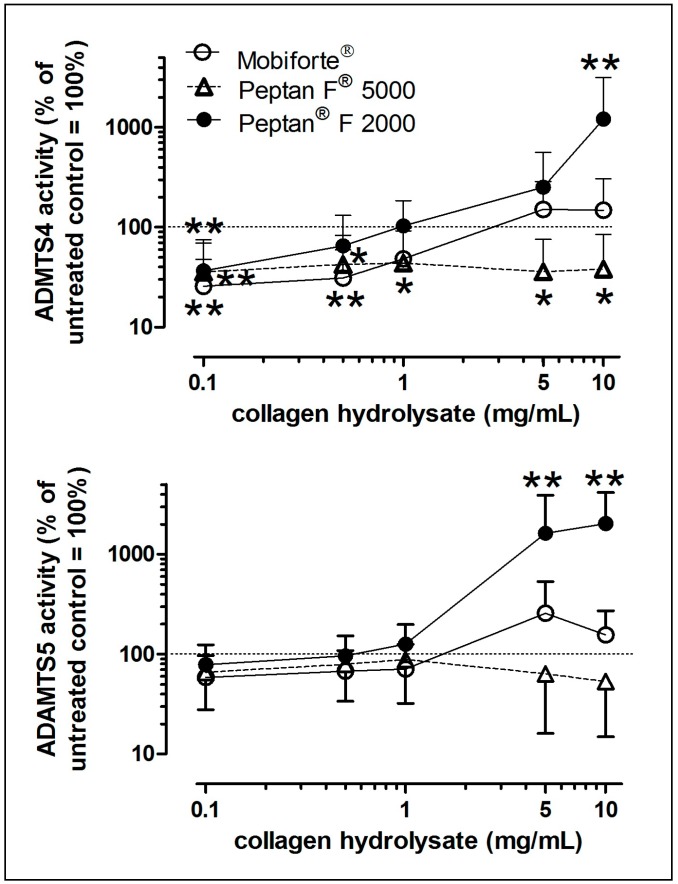
Concentration-dependent effects of CHs on the activities of aggrecanases. The activities of rhADAMTS4 (**A**) and rhADAMTS5 (**B**) in the presence or absence of 0.1, 0.5, 1, 2 and 10 mg/mL of Mobiforte^®^, Peptan^®^ F 5000 or Peptan^®^ F 2000 were determined using the rhAggrecan-IGD as a substrate. The activities of both aggrecanases are expressed as the percentage of untreated controls (=100%). Data are shown as the mean + SD (*n* = 4–6). Significant differences between treated and untreated controls (100%) were defined with p-values as follows: * 0.01< *p* ≤ 0.05, ** 0.001 < *p* ≤ 0.01.

To examine the activity of the two aggrecanases that mediate the normal and pathophysiological turnover of proteoglycan, we performed an in vitro assay with recombinant human aggrecan interglobular domain (rhAggrecan-IGD) as the substrate. The activity of ADAMTS4 was significantly inhibited by the three CHs at lower concentrations (Figure 5A). However, at higher concentrations (5 and 10 mg/mL), Peptan^®^ F 2000 stimulated ADAMTS4, whereas Peptan^®^ F 5000 still significantly inhibited it.

A similar bell-shaped concentration-dependent response to Peptan^®^ F 2000 was observed for ADAMTS5: at 2 and 10 mg/mL, its activity increased by 10-fold and 20-fold, respectively (Figure 5B). However, Peptan^®^ F 5000 and Mobiforte^®^ and Peptan^®^ F 2000 (at 1 mg/mL or lower) did not affect the activity of ADAMTS5 (Figure 5B).

### 2.7. Levels of Catabolic MMPs and TIMPs

**Figure 6:**
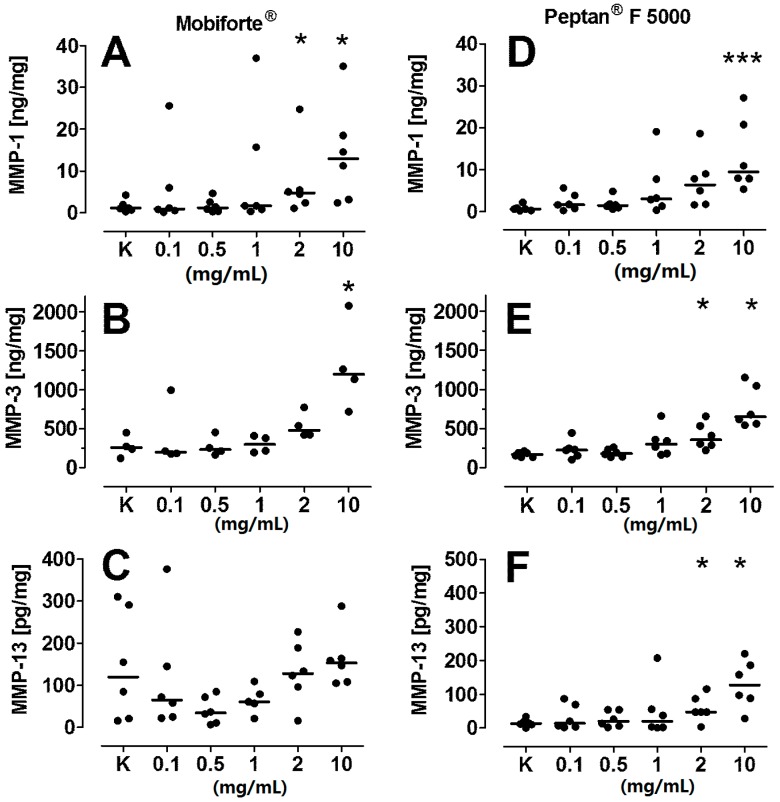
Concentration-dependent effects of CHs on the levels of MMP-1, MMP-3 and MMP-13. MMPs were determined within nutrient media of cultured human articular cartilage explants. Following stabilization of explant metabolism for 4–6 days, explants were treated for an additional six days with 0–10 mg/mL CH. MMP-1 (**A**,**D**); MMP-3 (**B**,**E**); and MMP-13 (**C**,**F**) were measured by ELISA, and data are shown as dot plots with their median (*n* = 6). Significant differences between treated and untreated controls (100%) were defined with *p*-values as follows: * 0.01 < *p* ≤ 0.05, *** *p* ≤ 0.001.

Because human type II collagen fragments from OA cartilage can stimulate a catabolic reaction [28,29], we examined to what extent CHs have similar effects. Mobiforte^®^ concentration-dependently elevated MMP-1 and -3 levels in the media by nine-fold and five-fold, respectively (Figure 6A,B). Furthermore, Peptan^®^ F 5000 significantly increased the amount of MMP-1 (18-fold), MMP-3 (5-fold) and MMP-13 (9-fold) at its highest concentration (Figure 6D–F). However, the levels of MMP-1, -3 and -13 remained unaffected by Peptan^®^ F 2000.

Further, the TIMP-1 and -3 levels were not influenced by any of the three CHs, tested between 0.1 and 10 mg/mL.

### 2.8. NO Production in Human Cartilage Explants

**Figure 7:**
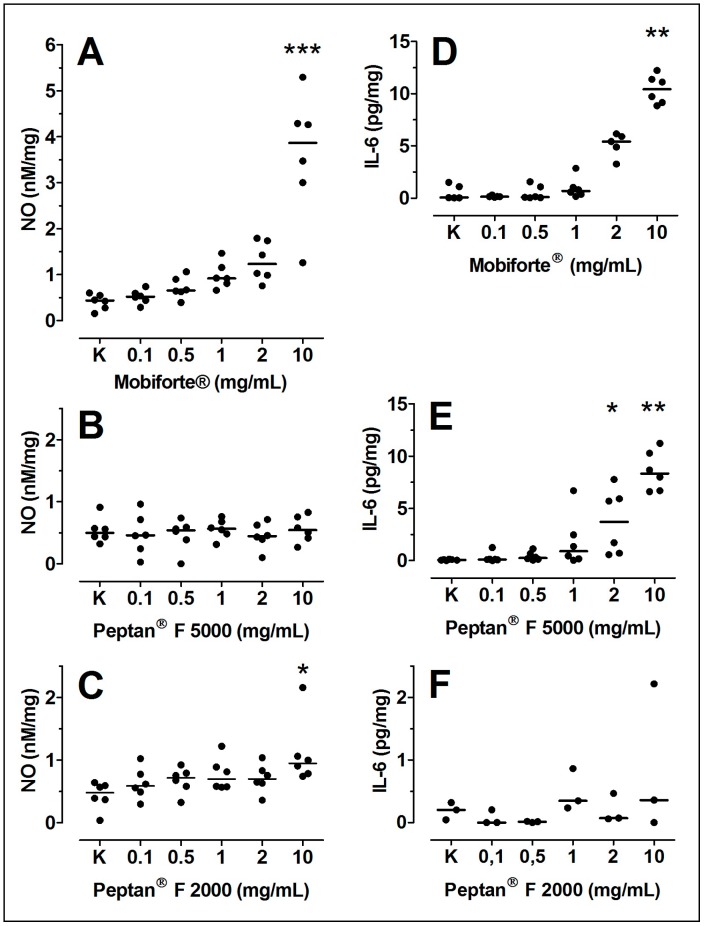
Concentration-dependent effects of CHs on NO and IL6. NO (**A**–**C**); and IL6 (**D**–**F**) were determined in the media of cultured articular cartilage explants treated with 0–10 mg/mL CHs. Data are shown as dot plots with their median (*n* = 6). Significant differences between treated and untreated controls (100%) were defined with p-values as follows: * 0.01 < *p* ≤ 0.05, ** 0.001 < *p* ≤ 0.01, *** *p* ≤ 0.001.

We analyzed the formation of NO, which affects the synthesis of proteoglycans and collagen as a significant inflammatory mediator. Mobiforte^®^ (9-fold) and Peptan^®^ F 2000 (3-fold) concentration-dependently increased the secretion of NO from the explants to the nutrient media (Figure 7A,C), whereas Peptan^®^ F 5000 (Figure 7B) had no effect.

### 2.9. Levels of IL-6, sIL-6R and sgp130 in Cultured Cartilage Explants

Because IL-6 is upregulated in OA cartilage [43], we measured IL-6 levels and its activity regulating sIL-6R and sgp130. Mobiforte^®^ and Peptan^®^ F 5000 concentration-dependently raised IL-6 levels by up to 23-fold and 114-fold, respectively (Figure 7D,E). The concentrations of sIL-6R and sgp130 were unaltered by any of the CHs.

### 2.10. Viability of Chondrocytes in Explants after CH Treatment

Because the three CHs differed in their peptide composition, we also quantified the viability of chondrocytes using cartilage sections that were stained with fluorescein diacetate and propidium iodide. We found that 10 mg/mL CH was not cytotoxic to chondrocytes in the superficial, intermediate and radial zones of explanted cartilage. Thus, we excluded the possibility that the metabolic results above were attributed to decreased chondrocyte viability.

## 3. Discussion

CHs have been used in a wide range of applications, such as pharmaceutical capsules, nutricosmetics, beverages, food, confectionery, dietary supplements and nutraceuticals. For several centuries, many health benefits for the skin, bone and joints have been claimed for collagen peptides, which are obtained as bioactive ingredients from native fish, bovine or porcine collagen [3,4,5,6,7,8,9,10]. Our study was motivated by the limited existing data on their safety, efficacy and mode of action, due in part to the lack of regulation of these nutritional compounds by stringent pharmaceutical drug laws.

In the search for CHs that stimulate the biosynthesis of and inhibit the degradation of extracellular matrix from human articular cartilage, we examined two fish- and one pig-derived CHs. In these novel findings, the three CHs: (a) differed in peptide composition; (b) did not induce the synthesis of type II collagen by human chondrocytes; and (c) had disparate effects on inflammatory mediators, major catabolic enzymes and their natural inhibitors from human articular cartilage.

### 3.1. Biochemical Characterization of CHs

By MALDI-TOF-MS analysis, we observed robust differences between Mobiforte^®^, Peptan^®^ F 5000 and Peptan^®^ F 2000. These CHs differed with respect to the total number of peaks, representing individual peptides in each preparation. We used the precise reflector mode in MALDI-TOF-MS, a mass gate setting of 500–4000 *m*/*z* and a high signal-to-noise ratio of >10 to identify peaks accurately. Further, although both Peptan^®^ preparations (obtained from fish) had approximately the same number of peptides, only six peptides were shared. The porcine CH Mobiforte^®^ had more than twice as many individual peptides, but fewer than 2% of peptides in common with the two Peptan^®^ preparations.

These mass spectrometry results were supported by our NMR measurements, clearly demonstrating the heterogeneous nature of the peptide mixtures in each CH preparation. The molecular weights of the CHs determined by NMR-DOSY differ from the corresponding data obtained by MALDI-TOF-MS and reported by the manufacturers. This is an important result since it indicates that the aggregation behavior of the CHs under study is clearly related to the chosen experimental conditions such as temperature, pH value and concentration. This finding could have a pharmacological impact. Furthermore, we have proven that it is possible to discriminate various CHs under defined experimental conditions just by diffusion parameters using NMR-DOSY spectroscopy.

In addition, none of the CHs contained collagenous peptides that were able to form amyloid fibrils, as demonstrated by ThT and AFM assays. The ability to form amyloid fibrils is a generic property of polypeptide chains, and most peptides and proteins have the potential to form such structures. It was found that amyloid formation can be seeded by a preformed amyloid fibril. Seeding is most likely the mechanism by which amyloid deposits spread in the human body. Therefore, it is of interest to know if collagen hydrolysates have the ability to form amyloid aggregates because they can induce cytotoxicity or act as seeds for amyloid aggregation of other poly/peptides.

Our results are consistent with our previous mass spectrometric analysis of three bovine CHs—RDH, Peptan^®^ B 5000; RDH-N, Peptan^®^ B 2000, and CH-Alpha^®^—for which we reported disparate peptide compositions for each preparation [2]. Thus, the expression “collagen hydrolysate” is a generic term of a heterogeneous group of nonfibrillating peptide mixtures from the collagen of various species and body parts through various production steps.

### 3.2. Collagen Biosynthesis of Human Cartilage as Modulated by CHs

CHs of bovine origin were reported to enhance collagen synthesis in young bovine chondrocytes [11]. Our study also analyzed collagen synthesis, using human articular knee cartilage. In our study, an advanced and sophisticated dual radiolabeling method was applied to examine the biosynthesis of type II collagen exclusively, representing approximately 90% of total cartilage collagen [2]. We did not observe any effects of CHs on the biosynthesis of collagen, even at previously-used concentrations [11]. Our data are consistent with our findings of a lack of an effect of bovine CHs on human cartilage collagen synthesis [2].

However, our data contrast those of Oesser et al. [11] who reported a stimulatory effect using bovine chondrocytes. These differences might be attributed to differences in the species, age and health status of the joints [41,42,44] and the analytical procedure. We used a sophisticated method to measure collagen biosynthesis, whereas measurements of the incorporation of radioactive proline into proteins without any further purification [11] do not reflect collagen biosynthesis exclusively. In summary, none of the six CHs (three species and three companies) that we have examined here and earlier [2] has the potential to stimulate collagen biosynthesis in human articular cartilage.

Even at 10 mg/mL, there was no stimulatory effect on collagen synthesis. According to the manufacturers, the recommended daily dose of the CHs is 10 g to receive the advertised health benefit for joints and bones. Thus, the blood levels of CHs, even when completely absorbed within a short time after oral administration, will be less than 2 mg/mL and most likely below 1 mg/mL. Unfortunately, our understanding of the plasma levels of each CH mixture with respect to its individual peptides is limited [45,46]. Thus, the concentrations that we chose encompass those that are assumed to be achieved in vivo. However, during absorption and circulation throughout the body, peptides will be metabolized further, such that only a small fraction of the original peptides and their metabolized species ultimately reach the joints.

We chose an experimental design to reduce variation in the data and to attribute the pharmacological activity solely to the collagenous agents. To this end, a nutrient media with an approved serum substitute was selected, and human cartilage explants were allowed to first stabilize their metabolism before being treated with the CHs for an extended period of six days. Notably, by laser capture microdissection, regional and OA stage-dependent differences in osteoarthritic cartilage have been reported [47]. Thus, our explants were reproducibly obtained from the same predefined anatomical area of lateral condyles, which were osteoarthritic within a small range, based on Collins scores.

### 3.3. Effects of CHs on Catabolic Enzymes and Proinflammatory Mediators

Various proteinases mediate the destruction of cartilage matrix, a hallmark of OA. For example, ADAMTS4 and ADAMTS5, subsequently replaced by digestion with MMP-3 and -13, can cleave several sites in the core protein of aggrecan, the major proteoglycan of articular cartilage [19,20,25,26,48]. Further, MMP-1, -3 and -13 can digest collagen type II that is unmasked by proteoglycans [19,20,48,49].

Thus, we examined certain members of the MMP and ADAMTS families. Only high concentrations of Mobiforte^®^ induced proteoglycan loss, which is accompanied by elevated NO and MMP-3 levels, but not with increased activities of either aggrecanase. In contrast, Peptan^®^ F 2000 had a bell-like effect on the activities of ADAMTS4 and ADAMTS5, but did not influence TIMP-1 and -3 levels. At high concentrations, this CH likely induced by an allosteric effect high activities of both aggrecanases, whereas it had an inhibitory effect at lower levels. However, the proteoglycan loss was not modulated by any of the Peptan^®^ preparations, although it initially requires active aggrecanases to proteolyze the aggrecan in the interglobular domain [48]. In this line, it was reported that the mRNA expression of ADAMTS4 and ADAMTS5 in bovine chondrocytes remained unaffected by an unspecified CH [10]. Altogether, our in situ results indicate that the peptides in Peptan^®^ F 2000 enhance the activities of ADAMTS4 and ADAMTS5 in vitro, but did not enter the extracellular matrix of cartilage explants sufficiently to activate both aggrecanases in situ. However, it remains to be determined whether low concentrations of the three CHs are useful inhibitors of pathologically elevated ADAMTS4 activity.

Experimental studies in mice have shown that CHs inhibit zymosan-induced ear skin inflammation and downregulate proinflammatory cytokines, such as IL-6, TNFα and sICAM-1 [50,51]. Thus, we also examined whether these agents modulate IL-6 trans-signaling. We found that at relatively high concentrations, Mobiforte^®^ and Peptan^®^ F 5000 (2 and 10 mg/mL, respectively) increase IL-6 levels in cultured articular cartilage. Our data are supported by Comblain et al. [10], who noted elevated levels of IL-6 in cultured human chondrocytes that were treated simultaneously with IL-1 and 4 µg/mL of an unspecified CH. As a proinflammatory cytokine, IL-6 synergizes with IL-1, TNFα and mechanical injury to induce the loss of extracellular matrix from articular cartilage [33,34,35,36,37]. Thus, it remains to be determined whether and to what extent CHs and IL-1, TNFα and mechanical injury cooperate during proteoglycan and collagen loss from articular cartilage.

This study was performed to identify the molecular mechanism of the chondroprotective activity of CHs, which has been proposed by other groups [11,52]. Our novel study presents three CH products from fish and pig, which differ significantly with regard to their content of collagen peptides and efficacy. The distinct activities on OA cartilage are attributed to the differences of the peptide composition, and the extent to which a single oligopeptide or a combination of oligopeptides, aggregates and metabolized peptides contributes in vivo to the major components of joint tissues remains to be determined.

## 4. Materials and Methods

### 4.1. MALDI-TOF Mass Spectrometric Analysis

Porcine CH (Mobiforte^®^, Astrid Twardy GmbH, Unterföhring, Germany) and fish CH (FGH, Peptan^®^ F 5000; FGH-N, Peptan^®^ F 2000 from Rousselot SAS, Puteaux, France) were used for our experiments. By MALDI-TOF mass spectrometry in reflector mode, the total number of peaks, representing individual peptides, and the number of common peptides between all CHs were estimated as follows. The CHs (10 mg/mL) were dissolved in 0.1% trifluoroacetic acid (TFA) and subjected to reversed-phase solid-phase extraction (DSC-18, Supelco, Bellefonte, PA, USA) to remove background interference and increase the sensitivity and accuracy. Peptides from the CHs were washed, eluted with 10 mL 0.1% TFA in 80% acetonitrile and lyophilized after centrifugation in a speed vacuum to remove excess acetonitrile. The samples were then redissolved and fractionated by reversed-phase high-performance liquid chromatography on a Waters XBridge™ C18 column (Eschborn, Germany) to obtain 15 fractions in 1 h. Fractions with collagenous peptides were lyophilized again and frozen at −20 °C until analysis by mass spectrometry.

Fractionated samples were redissolved in 0.1% TFA and mixed with 2.5-dihydroxybenzoic acid and methylendiphosphonic acid (5 mg/mL each) as the matrix solution and with an internal peptide calibration standard. These solutions were applied to the MALDI-TOF target as 2-µL droplets and allowed to crystallize. All mass spectra were acquired on a Bruker Ultraflex TOF/TOF MALDI instrument (Bruker Daltonics, Bremen, Germany) in positive ion mode. The system uses a pulsed nitrogen laser, emitting at 337 nm. The “low mass gate” was set to open at *m*/*z* = 500, and the upper mass limit was set to close at *m*/*z* = 4000 for the reflector mode. The extraction voltage was 25 kV.

Each mass spectrum was obtained as an average from approximately 350–750 single laser shots. The signal-to-noise ratio was set to >10 to avoid any artifact measurements. Each CH was measured in independent replicates (*n* = 3), and common peaks that represented individual peptides between all 3 replicate measurements were used to determine the total number of peptides per preparation or to compare the 3 CHs. Only peaks with a mass deviation of <50 ppm in the 3 replicate measurements were considered to be identical.

The mean molecular weight was obtained in linear mode and calculated from the *m*/*z* value in the middle between those of the ascending and descending peaks, reaching 50% of the peak maximum.

### 4.2. Nuclear Magnetic Resonance Spectroscopy

One- and 2-dimensional proton NMR spectra can be used to describe various CHs with respect to their chemical shift patterns. With 2-dimensional TOCSY NMR, it is possible to analyze the magnetization transfer by the chemical bonds and obtain characteristic signal patterns [2].

The CHs were dissolved at 3 mg in 0.5 mL water (90% H_2_O/10% D_2_O). The NMR experiments were performed on a 600-MHz Bruker Avance III spectrometer at 298 K. The 2D-TOCSY experiments (DIPSI-2; mixing time 80 ms) were recorded with 512 (F1) × 1024 (F2) complex data points and a spectral width of 7212 Hz (12 ppm). Water suppression was performed using excitation sculpting, and 16 scans per increment were accumulated with an inter-scan recovery delay of 1.5 s. For processing, we used zero-filling to 1024 (F1) × 2048 (F2) data points prior to Fourier transformation, followed by baseline correction in both dimensions. Spectra were calibrated against internal water. DOSY (Diffusion-Ordered Spectroscopy) NMR experiments were also performed using 3 mg CH in 0.5 mL water (90% H_2_O and 10% D_2_O). The built-in software from Bruker and mDOSY/CONTIN was used for inverse Laplace transformations.

### 4.3. Amyloid Fibrillization and Atomic Force Microscopy of CHs

All 3 types of CHs were dissolved in 20 mM Na_2_HPO_4_ + 80 mM NaCl buffer, pH 6.0, to a final concentration of 10 mg/mL prior to a 2-h incubation at 65 °C with constant stirring (1200 rpm) using magnetic stirrers. Aliquots (10 µM) were withdrawn from stock solutions at certain intervals and measured by thioflavin T (ThT) fluorescence assay and atomic force microscopy.

For the ThT fluorescence assay, ThT solution was added to the samples with 10 µM CHs at a protein:ThT ratio of 1:2. Measurements were made on a Synergy MX (BioTek Germany, Bad Friedrichshall, Germany) spectrofluorometer in a 96-well plate. The excitation wavelength was set to 440 nm, and the emission was recorded at 485 nm. The excitation and emission slits were adjusted to 9.0/9.0 nm, and the top probe vertical offset was 6 nm.

For atomic force microscopy, 10 µM CH samples were placed dropwise on the freshly-cleaned surface of the mica. After 10 min of adsorption, samples were washed with MilliQ water and dried under nitrogen gas. Images were taken in tapping mode using a scanning probe microscope (Veeco di Innova, Bruker AXS Inc., Madison, WI, USA) that was equipped with an NCHV cantilever (Bruker AXS Inc., Madison, WI, USA), which had a specific resistance of 0.01–0.025 Ω·cm, antimony (*n*)-doped Si, a tip curvature radius of 10 nm at a scan rate of 0.25–0.5 kHz and a resolution of 512 pixels per line (512 × 512 pixels/image). No smoothing or noise reduction was applied.

### 4.4. Specimen Selection

Articular cartilage was obtained at full thickness from the lateral femoral condyles of OA patients who were undergoing knee replacement surgery (collagen biosynthesis experiments: *n* = 4–5, age 61.2 + 7.7 years, BMI 32.0 + 3.2; cartilage degradation experiments: *n* = 6, age 73.3 + 5.6 years, BMI 30.7 + 3.7). Some OA patients had comorbidities, such as arterial hypertension (8×), diabetes mellitus (1×) and hypothyrosis (1×). The degree of changes in femoral condyles in OA was then determined per Collins [53]. Four-millimeter-diameter cartilage discs were washed 3 times with Gey’s Balanced Salt Solution (GBSS) and cultured as described below. The time that was needed to process cartilage that was obtained from surgery for culture never exceeded 4 h.

OA patients were selected at random from our clinic. The use of human articular cartilage for this study was approved by the local ethics committee of the Justus Liebig University Giessen (Az 106/03), and all patients provided written informed consent before the experiments were begun.

### 4.5. Articular Cartilage Explant Culture

Cartilage explants from moderately (Collins Grade 1.5–3) affected lateral human OA condyles were cultured separately in 2.0 mL Ham’s F-12, 2.5 mM HEPES (pH 7.2), containing 30 µg/mL alpha-ketoglutarate, 300 µg/mL glutamine, 50 µg/mL ascorbate, 1.0 mM Na_2_S0_4_, 20 units/mL penicillin, 10 µg/mL streptomycin, 2.5 µg/mL amphotericin B, 50 µg/mL gentamycin, 485 µg/mL CaCl_2_ dihydrate, and 1% (*v*/*v*) CR-ITS^+TM^ Premix (Collaborative Biomedical Products, Bedford, MD, USA) [2,54]. Explants were cultured for 4–6 days in a normal benchtop CO_2_ incubator under sterile conditions to first stabilize metabolism of the cartilage at 37 °C, 5% CO_2_ and 95% relative humidity.

In a separate set of experiments, collagen biosynthesis was examined using a novel dual radiolabeling procedure that was developed by Goodwin et al. [55] and has been described in detail [2]. Explants from 4–5 patients were used to analyze collagen synthesis (*n* = 4–5). Briefly, radioactive hydroxyproline was isolated to minimize the heterogeneity between pairs of samples, using [^3^H]-proline incorporation as a baseline measurement and [^14^C]-proline incorporation during the experimental treatment, such that each explant has its own internal control.

In a separate set of experiments, cartilage degradation was analyzed. Only lateral condyles of patients with Collins Grade 1.5–3 cartilage were used, and the metabolism of cultured explants was first stabilized for 4–6 days. Explants from 6 patients were cultured for 6 days in the presence of 0–10 mg/mL CH, with a change in media after 3 days, and the loss of proteoglycans, MMPs, TIMPs, NO, IL-6, sIL-6R, and sgp130 from explants into the nutrient media was measured. Media and explants were frozen at −20 °C in the presence of proteinase inhibitors until analysis.

### 4.6. Analysis of Collagen Synthesis

Collagen biosynthesis was analyzed by isolating radioactive hydroxyproline that was derived exclusively from collagen, whereas radioactive proline was found in all proteins per a novel procedure that was developed by Goodwin et al. [55] and has been described in detail [2]. Preliminary experiments demonstrated that unincorporated radioactive precursors were removed completely by this procedure. The radioactivity of [^3^H]- and [^14^C]-labeled hydroxyproline oxidation products was normalized with respect to the dry weights of the explants. [^3^H]-radioactivity was used as an internal control by first calculating the ^14^C/^3^H ratio of the explants, as described by Goodwin et al. [55]. This ratio was then used to determine the percentage of collagen synthesis of treated explants versus untreated (=100%) controls. Thus, the untreated controls provided a baseline against which the treated explants were compared [2].

### 4.7. Analysis of Proteoglycan Loss

Papain-digested cartilage explants and culture media were assayed for sulfated glycosaminoglycans (GAGs) by reaction with 0.25 mL 1,9-dimethylmethylene blue dye solution (Serva, Heidelberg, Germany) in polystyrene 96-well plates and quantified by spectrophotometry at 523 nm on an ELISA plate reader [54]. Chondroitin sulfate A from bovine trachea (Sigma, St. Louis, MO, USA) was used as the standard as already detailed [56]. Proteoglycan loss was then calculated, based on the ratio of GAGs in the media to all GAGs in the media plus explants. The data were normalized to the cartilage wet weight.

### 4.8. Aggrecanase Activity

The activity of rhADAMTS4 and rhADAMTS5 in the presence and absence of 0–10 mg/mL CH was determined in vitro using a recombinant human fragment of the interglobular domain of aggrecan (rhAggrecan-IGD; MD Biosciences, Egg, Switzerland) as the substrate, as detailed [54]. Briefly, after proteolytic cleavage of rhAggrecan-IGD by 7.5 nM rhADAMTS4 (F213-C685 from R&D Systems, Wiesbaden, Germany) or 7.5 nM rhADAMTS5 (S262-P622 from R&D Systems), an aggrecan peptide with the N-terminal sequence ARGSVIL was released and quantified using 2 monoclonal antipeptide antibodies (MD Biosciences). The % inhibition was calculated, based on a standard curve that was generated with untreated enzymes (*n* = 4–6).

### 4.9. Levels of Catabolic MMPs and TIMPs

MMP-1, -3 and -13 and TIMP-1 and -3 levels in the media were measured using commercially available ELISA kits (MMP-1 kit from Calbiochem, Darmstadt, Germany; TIMP-1-, MMP-3- and MMP-13 kits from GE Healthcare, Little Chalfont, UK; TIMP-3 kit from Biospes, Chongqing, China) per the manufacturer´s instructions. Preliminary experiments revealed that none of the CHs interfered with the ELISAs. The data were normalized to the cartilage wet weight.

### 4.10. NO Production

Nitrite levels in the media were measured in duplicate by Griess reaction with sodium nitrite as the standard as described [54,57]. Briefly, nitrate in 0.1 mL media was first reduced at 37 °C for 20 min with 10 µL nitrate reductase (0.4 U/mL, Roche Diagnostics GmbH, Mannheim, Germany). Media samples were then mixed with an equal volume of Griess reagent (1% sulfanilamide and 0.1% *N*-1-naphthylethylenediamine dihydrochloride in 25% (*v*/*v*) H_3_PO_4_) and incubated for 5 min at room temperature, and the optical density was measured at 523 nm on an ELISA photometer [54,57]. The data were normalized to the cartilage wet weight.

### 4.11. Analysis of IL-6, sIL-6R and sgp130

IL-6, sIL-6R and sgp130 were measured in the media using commercially available ELISA kits (IL-6 kit from eBioscience, Vienna, Austria; sgp130 and sIL-6R kits from R&D, Abingdon, UK) per the manufacturer’s instructions.

### 4.12. Chondrocyte Viability

The viability of chondrocytes in each anatomical zone of the cartilage explants (superficial, intermediate and deep layer) was determined in slices of separately cultured explants that were treated with or without 10 mg/mL CH using fluorescein diacetate (Sigma, St. Louis, MO, USA) and propidium iodide (Sigma, St. Louis, MO, USA) as described [2,54]. Briefly, 3 slices per explant, at least 50 viable or dead cells, as indicated by green or red fluorescence, were counted at 2 sites in each of the 3 cartilage layers under a fluorescence microscope at 200-fold magnification.

### 4.13. Statistical Analyses

Each experiment was repeated several times in explants from 4–5 patients to analyze collagen synthesis (*n* = 4–5) and in explants from 6 additional patients to measure cartilage degradation (*n* = 6). The data from the treated explants were compared with values from untreated controls from the same joint. Groups of data were analyzed by Friedman test, followed by Dunn’s multiple comparison test. Data are presented as the mean + standard deviation (Figure 3A, Figure 4 and Figure 5) or as a dot plot with median values (Figure 6 and Figure 7), and the significance was set to *p* ≤ 0.05.

## 5. Conclusions

Our study can be translated to the clinical situation only with some restrictions. As such, we observed in vitro effects only at relatively high concentrations, which might be considered protective or, in most cases, detrimental to cartilage. Furthermore, there are other pathways and enzymes, such as cathepsins B and S, which are involved in cartilage degradation [58]. Further, OA is no longer viewed as a focal cartilage disease, but is considered as a low-level inflammatory disease that affects the entire joint or body [25,26,30]. Thus, CHs might also influence other cells of the joint, such as bony cells and synovial fibroblasts, meriting further study.

Based on our current and earlier [2] in vitro findings, we conclude that: (a) the term “collagen hydrolysate” is the generic name of a heterogeneous group of nonfibrillating collagenous peptide mixtures; and (b) CHs do not stimulate type II collagen biosynthesis in human articular cartilage. Further, due to the high variability in peptide composition between CH preparations, no effect can be extrapolated from a CH to another mixture. Thus, each orally-administered CH preparation must be carefully analyzed in vitro and in vivo regarding pleiotropic effects before this peptide mixture can be attested to be an effective and safe nutraceutical for patients.

## Abbreviations

OA	Osteoarthritis
CH	Collagen Hydrolysate
MS	Mass Spectrometry
MMP	Matrix Metalloproteinase
ADAMTS	A Disintegrin and Metalloproteinase with a Thrombospondin Motif
TIMP	Tissue Inhibitor of Metalloproteinases
IL	Interleukin
sIL-6R	Soluble Interleukin-6 Receptor
sgp 130	Soluble Glycoprotein 130
MALDI-TOF	Matrix-Assisted Laser Desorption/Ionization-Time of Flight
TFA	Trifluoroacetic Acid
TOCSY	Total Correlation Spectroscopy
NMR	Nuclear Magnetic Resonance
ThT	Thioflavin T
BMI	Body Mass Index
DOSY	Diffusion Ordered Spectroscopy
